# Фульминантный гиперкортицизм вследствие АКТГ-продуцирующей феохромоцитомы

**DOI:** 10.14341/probl13374

**Published:** 2023-11-02

**Authors:** З. Т. Усеинова, Е. А. Пигарова, Д. Г. Бельцевич, А. Шевэ, Л. К. Дзеранова, И. И. Ситкин, Н. В. Тарбаева, А. В. Хайриева, М. В. Дегтярев, Н. М. Платонова, Е. А. Трошина, Е. В. Бондаренко

**Affiliations:** Национальный медицинский исследовательский центр эндокринологии; Национальный медицинский исследовательский центр эндокринологии; Национальный медицинский исследовательский центр эндокринологии; Национальный медицинский исследовательский центр эндокринологии; Национальный медицинский исследовательский центр эндокринологии; Национальный медицинский исследовательский центр эндокринологии; Национальный медицинский исследовательский центр эндокринологии; Национальный медицинский исследовательский центр эндокринологии; Национальный медицинский исследовательский центр эндокринологии; Национальный медицинский исследовательский центр эндокринологии; Национальный медицинский исследовательский центр эндокринологии; Национальный медицинский исследовательский центр эндокринологии

**Keywords:** АКТГ-эктопированный синдром, феохромоцитома, синдром гиперкортицизма

## Abstract

Эндогенный гиперкортицизм (ЭГ) — это тяжелый симптомокомплекс, вызываемый избытком глюкокортикоидов в организме. В соответствии с этиологией выделяют АКТГ-зависимый и АКТГ-независимый варианты, которые, согласно литературе, встречаются в 70–80% и 20–30% случаев соответственно. Редкой причиной АКТГ-зависимого эндогенного гиперкортицизма является АКТГ-эктопированный синдром (АКТГ-ЭС) (около 15–20% случаев). АКТГ-ЭС — синдром гиперпродукции адренокортикотропного гормона (АКТГ) нейроэндокринными опухолями внегипофизарного происхождения. АКТГ могут секретировать различные опухоли: бронхолегочный карциноид, мелкоклеточный рак легкого, реже — карциноид тимуса, островковоклеточные опухоли и карциноид поджелудочной железы, медуллярный рак щитовидной железы, карциноидные опухоли кишечника, яичников, а также феохромоцитома (ФХЦ).

В данной публикации представлен клинический случай редко выявляемой паранеопластической продукции АКТГ феохромоцитомой. Пациентка имела клинические проявления гиперкортицизма, в связи с чем обратилась в ГНЦ РФ ФГБУ «НМИЦ эндокринологии» Минздрава России. В ходе обследования подтвержден синдром Кушинга (СК), при мультиспиральной компьютерной томографии (МСКТ) брюшной полости выявлено объемное образование левого надпочечника. Дополнительное обследование зафиксировало многократное повышение уровня метилированных производных катехоламинов в моче. Впоследствии больной была проведена левосторонняя адреналэктомия. Диагноз «Феохромоцитома» подтвержден морфологически, иммуногистохимическое исследование продемонстрировало интенсивную экспрессию хромогранина А и АКТГ-клетками опухоли.

## Abstract (en)

Endogenous hypercorticism (EH) is a severe symptom complex caused by hypercortisolemia; according to the etiology, ACTH-dependent and ACTH-independent variants are distinguished, which, according to the literature, occur in 70–80% and 20–30% of cases, respectively. A rare cause of ACTH-dependent endogenous hypercorticism is ACTH-ectopic syndrome (ACTH-ES) (about 15-20% of cases). ACTH-ES is a syndrome of adrenocorticotropic hormone (ACTH) hyperproduction by neuroendocrine tumors of extrahypophyseal origin. Various tumors can secrete ACTH: bronchopulmonary carcinoid, small cell lung cancer, less frequently, thymus carcinoid, islet cell tumors and pancreatic carcinoid, medullary thyroid cancer, carcinoid tumors of the intestine, ovaries, as well as pheochromocytoma (PCC).

This publication presents a clinical case of rarely detected paraneoplastic ACTH production by pheochromocytoma. The patient had clinical manifestations of hypercorticism, therefore, she applied to the Russian National Research Center of Endocrinology of the Ministry of Health of Russia. During the examination Cushing’s syndrome (CS) was confirmed, multispiral computed tomography (MSCT) of the abdominal cavity revealed a voluminous formation of the left adrenal gland. Additional examination recorded a multiple increase in urinary catecholamine levels. Subsequently, the patient underwent left-sided adrenalectomy. The diagnosis of pheochromocytoma was confirmed morphologically, immunohistochemical study demonstrated intensive expression of chromogranin A and ACTH by tumor cells.

## АКТУАЛЬНОСТЬ

Синдром эктопической секреции АКТГ является редкой патологией в практике врача эндокринолога и вместе с тем клинически тяжело протекающим заболеванием, приводящим к смертельному исходу при отсутствии своевременной диагностики и лечения. Согласно различным источникам литературы, АКТГ-эктопированный синдром встречается в 15–20% случаев диагностированного АКТГ-зависимого варианта ЭГ. В структуре АКТГ-эктопированного синдрома ФХЦ, по данным клинических исследований, составляет от 2,8 до 5,6%, тогда как бронхиальный карциноид встречается у 5–38,9% исследуемых, мелкоклеточный рак легких — у 3,3–50%, нейроэндокринная опухоль (НЭО) кишечника — 8,9–17%, карциноид тимуса — 4,7–11%, медуллярный рак щитовидной железы — 2–8,5% [1]. В этиологической структуре данного синдрома, наряду с медуллярным раком щитовидной железы, ФХЦ занимает одно из последних мест, что может затруднять диагностику и вводить в заблуждение даже опытного врача. Основная сложность заключается в топическом обнаружении очага аберрантной продукции АКТГ. В настоящее время при сомнительных результатах топической диагностики (малый размер/отсутствие аденомы гипофиза) существует возможность проведения селективного забора крови из нижних каменистых синусов — метода, обладающего высокой чувствительностью и специфичностью (94–96% и 100% соответственно) и направленного на дифференциальную диагностику болезни Иценко-Кушинга (БИК) с АКТГ-эктопией.

В связи с тем, что пациенты с подобной патологией нуждаются в более широком спектре диагностических исследований и незамедлительном лечении с учетом фульминантного и прогрессирующего течения, считаем важным поделиться собственным опытом ведения пациента с диагнозом «АКТГ-продуцирующая феохромоцитома».

## ОПИСАНИЕ СЛУЧАЯ

Пациентка Н., 31 год, обратилась в ГНЦ РФ ФГБУ «НМИЦ эндокринологии» Минздрава России с жалобами на общую и мышечную слабость, выраженную потливость, снижение памяти, нарушение речи, повышение уровня глюкозы капиллярной крови максимально до 9,7 ммоль/л, избыточную жажду, повышение уровней артериального давления максимально до 200/100 мм рт.ст., изменение внешности (округление лица, появление темных волос на лице, сыпи в области лица и зоны декольте), судороги в нижних конечностях.

Согласно анамнезу, пациентка считает себя больной с лета 2021 г., когда стала отмечать повышение АД до 200/100 мм рт.ст. Эпизоды повышения АД связывала с постоянным стрессом после рождения первого ребенка (2019 г.). Медикаментозная терапия не назначалась. Самочувствие пациентки оставалось удовлетворительным до осени 2022 г. 24.09.2022 пациентка перенесла приступ с потерей памяти, нарушениями речи. 28.09.2022 госпитализирована в областную клиническую больницу. В ходе госпитализации заподозрена болезнь Иценко-Кушинга/АКТГ-эктопированный синдром. По данным лабораторных исследований: кортизол — 1178,0 нмоль/л (171–536), АКТГ — 168,7 пг/мл (5,0–60,0), ТТГ — 1,25 мкМЕ/мл (0,27–4,2), св.Т4 — 14,1 пмоль/л (12,0–22,0), пролактин — 122,0 мМЕ/л (127–637), альдостерон — 93,2 пг/мл (0–199); ночной подавляющий тест с 1 мг дексаметазона — кортизол в 08:00 — 892,8 нмоль/л (норма — менее 50). Гликемия перед едой — 7,65–9,76 ммоль/л. По данным МРТ головного мозга, очаговых патологических изменений не выявлено, на МСКТ надпочечников — сегментарная гиперплазия левого надпочечника.

Госпитализирована в ГНЦ РФ ФГБУ «НМИЦ эндокринологии» Минздрава России для обследования и определения дальнейшей тактики лечения. При поступлении: состояние средней тяжести. Отмечается изменение внешности: лунообразное лицо, матронизм, перераспределение подкожно-жировой клетчатки по кушингоидному типу — сглаживание очертаний талии, истончение жировой клетчатки и кожи верхних и нижних конечностей, гирсутизм (рост жестких темных волос в области подбородка и над верхней губой), пустулезная сыпь в области шеи, зоны декольте, плечевого пояса и верхней половины спины. АД — 180/90 мм рт.ст., ЧСС — 89 уд/мин.

В ходе обследования в стационаре подтвержден АКТГ-зависимый гиперкортицизм. Данные гормональных исследований представлены в таблице 1.

**Table:** Таблица 1. Данные лабораторных исследований, выполненных при исходном обследовании и после консервативного лечения

Показатель	При исходном обследовании	3 нед. после инъекции ланреотида, 2 нед. после приема кетоконазола	6 месяцев после операции	Референсные значения
АКТГ утро (пг/мл)	313	243	51,02	7,2–63,3
АКТГ вечер (пг/мл)	204	-	14,47	2–25,5
Кортизол утро (нмоль/л)	2588	4933	444,4	171–536
Кортизол вечер (нмоль/л)	1548	-	-	64–327
Кортизол свободный в вечерней слюне (нмоль/л)	220	-	0,5	0,5–9,65
Кортизол свободный суточной мочи (нмоль/сут)	12 332	>35 000	111,8	100–379
Метанефрин суточной мочи (мкг/сут)	1122,9	-	83,81	25–312
Норметанефрин суточной мочи (мкг/сут)	1039,11	-	146,37	35–445
Хромогранин А (нмоль/л)	3,50	-	0,7	<2
Глюкоза венозной плазмы, натощак (ммоль/л)	11,34	-	4,5	3,1–6,1
Гликированный гемоглобин (%)	7,4	-	5,2	4–6
Калий (ммоль/л)	3,5	-	5,2	3,5–5,1

В биохимическом анализе крови зарегистрирована тяжелая гипокалиемия: калий — 1,9 ммоль/л (3,5–5,1). Пациентка ощущала выраженную мышечную слабость, тахикардию, периодические болезненные судороги в мышцах нижних конечностей. В рамках симптоматического лечения проводилась инфузионная терапия 4% раствором калия хлорида в объеме 240 мг/сут в течение недели, инициирован прием калий-сберегающих диуретиков (спиронолактон 300 мг/сут) и калий-содержащих таблетированных препаратов (Калинор: калия цитрат 1,56 г 3 раза в сутки), на фоне чего удалось постепенно нормализовать и стабилизировать калий крови на уровне: 3,5–4,59 ммоль/л. На фоне коррекции уровня калия крови несколько уменьшились проявления мышечной слабости, но выраженные болезненные судороги ног оставались, что требовало применения симптоматической терапии НПВС.

При скрининге осложнений гиперкортицизма, на основании полученных показателей (глюкоза венозной плазмы крови натощак, гликированный гемоглобин — указаны в табл. 1), пациентке диагностирован сахарный диабет вследствие эндогенного гиперкортицизма, в связи с чем инициирована инсулинотерапия в базис-болюсном режиме, достигнуты целевые показатели гликемии.

При установке АКТГ-зависимого характера ЭГ для дифференциальной диагностики БИК и АКТГ-эктопированного синдрома проведено МРТ-исследование головного мозга с контрастированием, которое выявило микроаденому задней части аденогипофиза размерами 3х3,5 мм (рис. 1).

**Figure:**
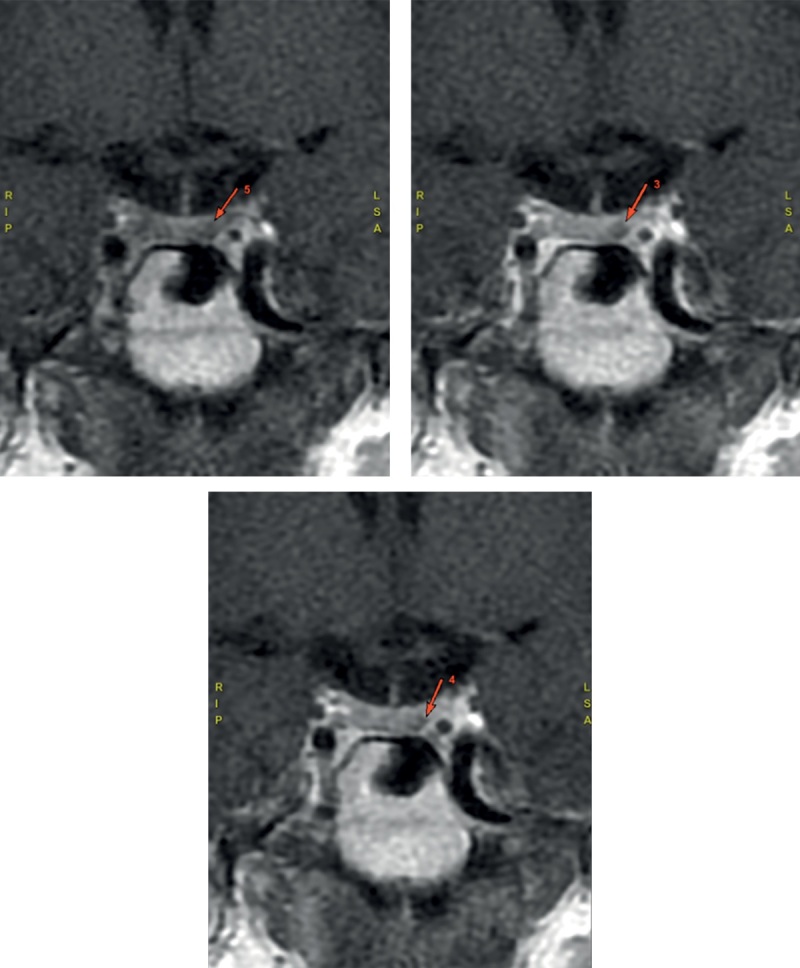
Рисунок 1. Магнитно-резонансная томография гипофиза — стрелками указана микроаденома задней части аденогипофиза.

Для определения источника секреции АКТГ пациентке выполнен двусторонний селективный забор венозной крови из нижних каменистых синусов (табл. 2), который показал отсутствие градиента секреции АКТГ: максимальный градиент справа составил 1,15, слева — 1,10. Анализ градиента пролактина и уровня постановки катетеров позволил исключить возможность технической ошибки.

**Table:** Таблица 2. Результаты забора крови из нижних каменистых синусов Максимальный градиент АКТГ между центром и периферией — 1,146 справа; АКТГ/пролактин — нормализованное соотношение составило: справа — 0,257, слева — 0,581. Результаты свидетельствовали в пользу внегипофизарного генеза гиперкортицизма.

АКТГ
Время	Правый синус	Левый синус	Периферия	Градиент АКТГ между центром и периферией	АКТГ/пролактин-нормализованное соотношение
Справа	Слева	Справа	Слева
-5 мин	270,2	244,6	235,7	1,146	1,038	0,257	0,581
0 мин	234,6	256,8	232,8	1,008	1,103
+3 мин	256,1	251,9	241,5	1,06	1,043
+5 мин	254,6	243,5	222,5	1,144	1,094
+10 мин	248	246,8	237,6	1,044	1,039
Пролактин
-5 мин	809,6	344,9	181,7	4,456	1,898	

Проведен поиск источника эктопии. МСКТ легких и средостения не обнаружило патологических образований. В свою очередь, МСКТ брюшной полости и забрюшинного пространства подтвердила образование левого надпочечника овальной формы (рис. 2) размерами 37х30х46 мм, злокачественного фенотипа (плотностью 42/49/69/60 Hounsfield units (HU) в нативную/артериальную/венозную/отсроченную (15 мин) фазы контрастирования соответственно, абсолютный индекс вымывания (APW) — 33%, относительный индекс вымывания (RPW) — 13%; нерезкая диффузная гиперплазия обоих надпочечников. Образование левого надпочечника расценено как очаг эктопической секреции АКТГ.

**Figure:**
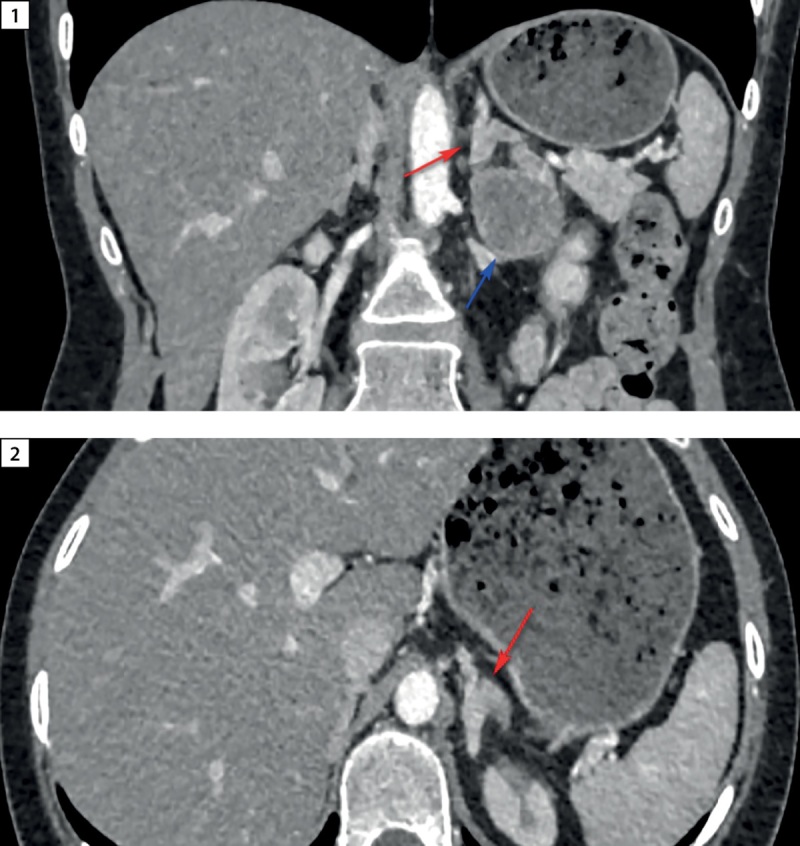
Рисунок 2. Визуализация левого надпочечника на МСКТ органов забрюшинного пространства: 1 — красная стрелка указывает на левый надпочечник, синяя стрелка — на опухолевое образование; 2 — красная стрелка указывает на левый надпочечник.

Учитывая наличие высокоплотной опухоли надпочечника, также принимая во внимание тот факт, что паранеопластическая секреция АКТГ может быть обусловлена наличием ФХЦ, проведено определение метилированных производных катехоламинов в суточной моче. По данным лабораторного анализа: метанефрин — 1122,9 мкг/сут (25–312), норметанефрин — 1039,11 мкг/сут (35–445), хромогранин А — 3,50 нмоль/л (<2 нмоль/л), что свидетельствовало о наличии ФХЦ.

В рамках предоперационной подготовки, для исключения возникновения неуправляемой гемодинамики при удалении хромаффинной опухоли, проводилось лечение альфа- и бета-адреноблокаторами (доксазозин 6 мг/сут, бисопролол 10 мг/сут).

Таким образом, ФХЦ левого надпочечника расценена как эктопический очаг секреции АКТГ.

Дополнительно проведена сцинтиграфия с Тс-99-Тектротидом — на полученных планарных сцинтиграммах (всего тела) достоверных признаков патологического очагового накопления РФП не определялось. При ОФЭКТ-КТ брюшной полости и частично органов грудной клетки: из латеральной ножки левого надпочечника исходит образование мягкотканной плотности размерами 39х30х49 мм, накапливающее РФП, что подтвердило умеренно-повышенную экспрессию соматостатиновых рецепторов в объемном образовании левого надпочечника (рис. 3).

**Figure:**
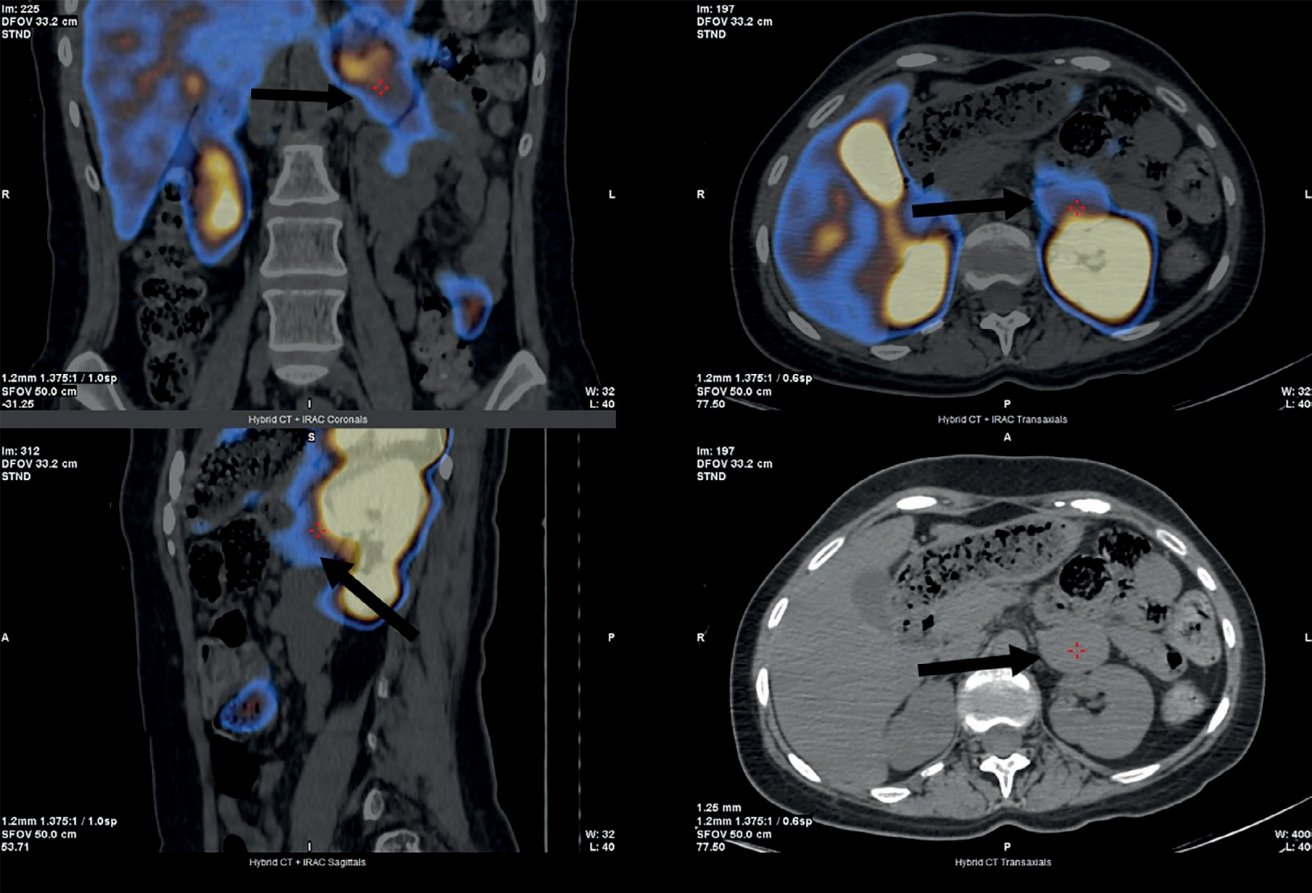
Рисунок 3. Соматостатин-рецепторная сцинтиграфия с Тс-99-Тектротидом в режиме «все тело», стрелочками указано новообразование левого надпочечника.

## Лечение

Учитывая тяжесть состояния пациентки, скорость прогрессирования гиперкортицизма, необходимость снижения проявлений осложнений ЭГ и полученных данных сцинтиграфии, произведена попытка воздействия на активность заболевания аналогом соматостатина пролонгированного действия Ланреотидом 120 мг подкожно — без эффекта, не возникло снижения секреции АКТГ или кортизола (результаты лабораторных исследований — табл. 1). В течение двух недель до оперативного вмешательства пациентка получала блокатор стероидогенеза — кетоконазол по 600 мг/сут, что также не оказало положительного влияния на уровень кортизола. В динамике наблюдения уровень свободного кортизола в суточной моче повышался в геометрической прогрессии с 12 332 до >35 000 нмоль.

У пациентки имелись жизненные показания к проведению оперативного лечения, обусловленные большими размерами и высокой плотностью образования левого надпочечника, а также выраженной сочетанной гормональной активностью (АКТГ+катехоламины). Пациентке проведена левосторонняя лапароскопическая адреналэктомия. Макро- и микропрепараты левого надпочечника с опухолью представлены на рисунке 4. В послеоперационном периоде надпочечниковая недостаточность не была зарегистрирована, однако зафиксировано падение уровня АКТГ (табл. 3), в связи с чем в течение первых 6 суток проводилась терапия глюкокортикостероидами (гидрокортизон 20 мг утром, 10 мг в обед, 10 мг в 16:00). Вследствие выраженной гипокалиемии (табл. 3) в послеоперационном периоде проводилась инфузионная терапия препаратами калия в сочетании со спиронолактоном 300 мг/сут. Результат калия перед выпиской — в пределах референса (4,43 ммоль/л). После операции отменена гормональная, гипотензивная, базис-болюсная инсулинотерапия в связи с достижением индивидуальных целевых значений АД и гликемии. Проводилась антибактериальная, противовоспалительная, обезболивающая, антитромботическая терапия. Выписана в удовлетворительном состоянии.

**Figure:**
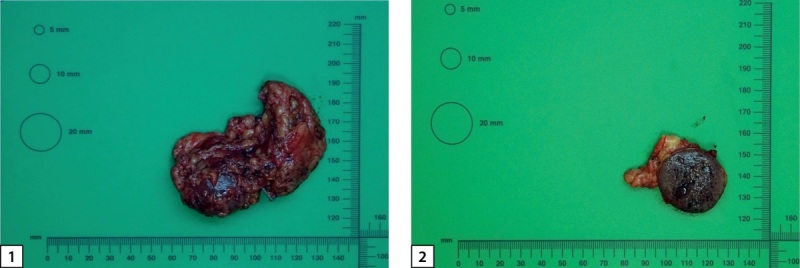
Рисунок 4. Макропрепарат левого надпочечника с опухолью: 1 — макровид левого надпочечника с опухолью, 2 — макровид левого надпочечника с опухолью на разрезе.

**Table:** Таблица 3. Данные лабораторных исследований, выполненных после операционного вмешательства

Показатель	1-е сутки	2-е сутки	3-и сутки	6-е сутки	6 месяцев	Референсные значения
АКТГ утро (пг/мл)	45,23	11,3	6,37	31,98	51,02	7,2–63,3
АКТГ вечер (пг/мл)	-	2,28	-	-	14,47	2–25,5
Кортизол утро (нмоль/л)	-	574,9	-	-	444,4	171–536
Кортизол свободный вечерней слюны (нмоль/л)	-	104,7	-	-	0,5	0,5–9,65
Калий	2,51	3,66	3,84	4,43	5,25	3,5–5,1

Удаленное новообразование левого надпочечника было представлено единым узлом буро-коричневого цвета диаметром до 3 см (рис. 4). При гистологическом исследовании определялась опухоль солидного и гнездного строения из хромаффинных клеток с оксифильной цитоплазмой. На 10 полей зрения выявлен 1 митоз (рис. 5). При проведении иммуногистохимического исследования в опухоли выявлена диффузная экспрессия хромогранина А, синаптофизина, АКТГ (рис. 6). Для оценки рецепторного статуса опухоли выполнена оценка экспрессии рецепторов соматостатина 2А типа (SSTR 2А) — 2 балла. Индекс пролиферации (Ki67) менее 5%. Учитывая результаты гистологического и иммуногистохимического исследований, поставлен диагноз феохромоцитомы, секретирующей АКТГ.

**Figure:**
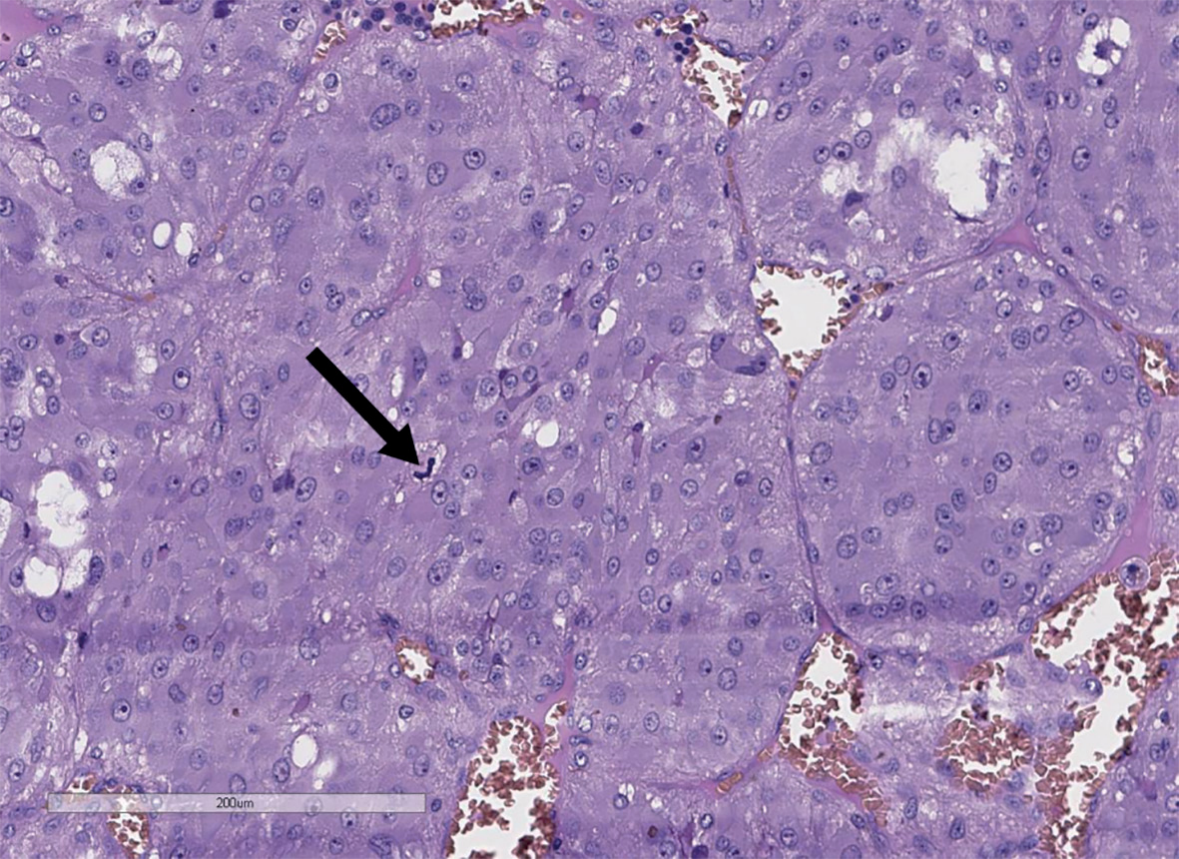
Рисунок 5. Гистологическое строение феохромоцитомы левого надпочечника (стрелкой показан митоз), гематоксилин-эозин, 200х.

**Figure:**
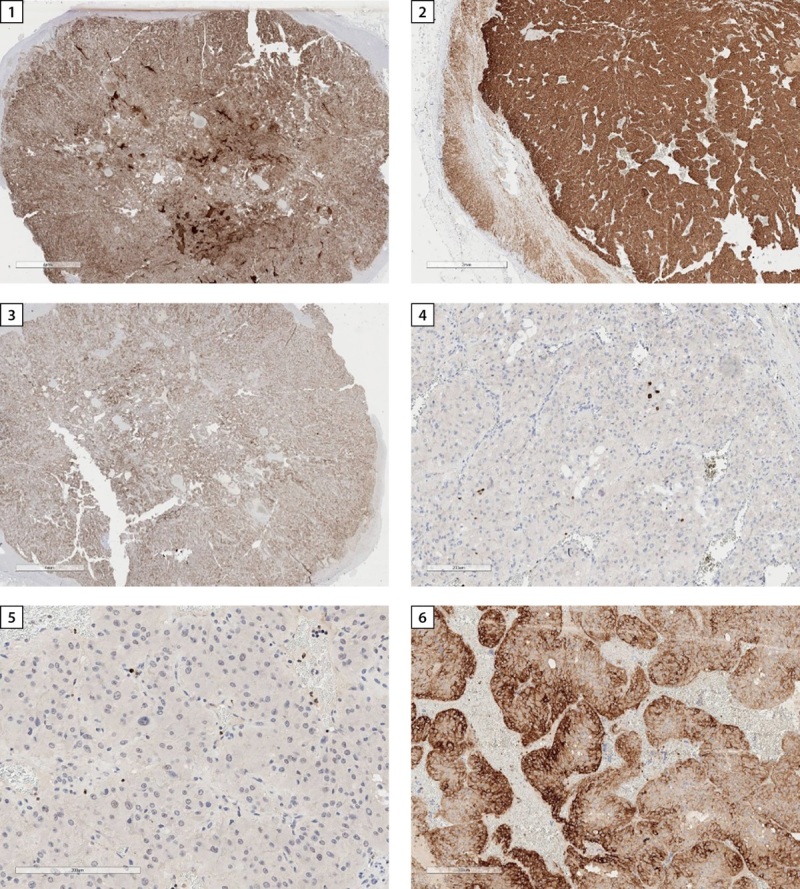
Рисунок 6. Иммуногистохимическое исследование: 1 — диффузная экспрессия хромогранина А, 4х; 2 — диффузная экспрессия синаптофизина, 2х; 3 — экспрессия АКТГ, 4х; 4 — уровень пролиферативной активности по экспрессии Ki67 составляет до 5%, 200х; 5 — при определении митотической активности с фосфогистоном определяется 4 митоза на 50 радиусе поля зрения (РПЗ), 200х; 6 — экспрессия рецепторов соматостатина 2А типа (SSTR 2A) — 2 балла, 300х.

Пациентке проведено полное секвенирование экзома на платформе Illumina методом парно-концевого чтения (2x150 п.о.), средняя глубина покрытия — 249x, процент целевых нуклеотидов с эффективным покрытием >10х — 99%. Однако в материале не обнаружено патогенных и вероятно патогенных вариантов, а также вариантов с неизвестной клинической значимостью, объясняющих причину заболевания на молекулярно-генетическом уровне.

При госпитализации в ГНЦ РФ ФГБУ «НМИЦ эндокринологии» МР через 6 месяцев после оперативного лечения отмечено обратное перераспределение подкожно-жировой клетчатки (исчезли надключичные подушечки, климактерический горбик, уменьшилась окружность живота), прекратился рост волос в области подбородка и над верхней губой, уменьшились проявления угревой сыпи, пациентка обратила внимание, что сыпь увеличилась в течение месяца после операции, а затем постепенно начала уменьшаться. Антигипертензивная терапия отменена в раннем послеоперационном периоде, потребность в сахароснижающей терапии посредством инсулина снизилась через 2 месяца, что потребовало отмены инсулинотерапии. Трудоспособность пациентки восстановилась через 2 месяца после оперативного вмешательства.

Лабораторное исследование показало нормализацию уровней АКТГ, кортизола и метилированных катехоламинов (табл. 1). По результатам МСКТ забрюшинного пространства с контрастным усилением, объемные образования не визуализировались: левый надпочечник удален, в ложе послеоперационные изменения, регионарные л/у не увеличены; правый надпочечник не утолщен, тело до 4 мм, ножка до 3 мм, дополнительных образований нет, регионарные л/у не увеличены.

Следовательно, у пациентки подтверждена клиническая и биохимическая ремиссия заболевания при отсутствии медикаментозной терапии.

## ОБСУЖДЕНИЕ

АКТГ-эктопированный синдром при ФХЦ — достаточно редкое явление, которое может привести к летальному исходу при отсутствии своевременной диагностики. Патогенез формирования АКТГ-эктопии не до конца изучен. Одна из причин возникновения эктопической секреции АКТГ в ФХЦ или в другой НЭО — активация некоторых генов. В частности, активация гена проопиомеланокортина (ПОМК) — предшественника АКТГ, что приводит к избыточной секреции последнего. Yuji Tani и соавт. в своем исследовании обнаружили увеличение экспрессии матричной рибонуклеиновой кислоты (мРНК) фермента РС2 в АКТГ-эктопических опухолях в 32 раза по сравнению с уровнем экспрессии при БИК [12]. Указанный фермент способствует синтезу α-меланоцитстимулирующего гормона из АКТГ, это приводит к кожной пигментации у данной категории пациентов, что, однако, отсутствовало у нашей пациентки. Наряду с вышеописанным исследователи отметили чрезмерную экспрессию мРНК гена IKZFI у пациентов с АКТГ-ЭС (экспрессия мРНК превышала в 65 раз таковую при БИК).

В патогенезе формирования ФХЦ различают два ведущих кластера генетических мутаций [15][16]. Первый кластер составляют мутации генов HIF2A, PHD2, VHL, SDHX, IDH, MDH2 и FH. Во втором кластере объединены генетические мутации, ассоциированные с нарушенной активацией сигнального пути киназы, как, например, мутации генов RET, NF1, KIF1Bβ, MAX и TMEM127 [14]. Исследователями были обнаружены мутации генов, предрасполагающие к развитию ФХЦ и параганглиомы. К ним относят мутации в генах GDNF, H-ras, K-ras, GNAS, CDKN2A, p53, BAP1 и BRCA 1 и 2 [8][15][16]. Соматические мутации были найдены только в гене HRAS [16], в свою очередь герминальные мутации выявлены в генах SDHA, SDHC, SDHAF2, FH, KIF1β и TMEM127 [8][15]. Однако, как указано выше, при полном секвенировании экзома нашей пациентки патогенных и вероятно патогенных вариантов не обнаружено.

Elliott PF и соавт. провели метаанализ АКТГ и/или КРГ-секретирующих феохромоцитом за период с 1966 по 2019 гг. [13]. Исследование включало в себя 99 случаев, среди которых 96 пациентов имели клинические и биохимические признаки гиперкортицизма. Среди клинических проявлений артериальная гипертензия имела место у 87 (93%), около половины пациентов имели сахарный диабет, примерно у четверти наблюдались симптомы психических нарушений. В дооперационном периоде медиана уровня АКТГ — 62,8 пмоль/л, медиана утреннего кортизола — 1399 нмоль/л, медиана суточной экскреции свободного кортизола с мочой — 5500 нмоль/сут. Более того, по результатам метаанализа, у 6 пациентов (12%) опухоли были диагностированы как часть синдрома множественных эндокринных неоплазий 2а.

За 30 лет практики в ГНЦ РФ ФГБУ «НМИЦ эндокринологии» МР находилось на стационарном лечении 6 больных с подобной патологией [4][5][7]. Длительность заболевания варьировала от 6 месяцев до 5 лет, при этом у всех отмечались следующие клинические симптомы манифестной формы СК: перераспределение подкожно-жировой клетчатки по кушингоидному типу, матронизм, «климактерический» горбик, надключичные подушечки, сахарный диабет, вызванный гиперкортицизмом, стероидная миопатия, артериальная гипертензия, вторичный остеопороз, гипокалиемия, у одной из пациенток наблюдалось резкое снижение иммунного статуса, проявляющееся рецидивирующим герпесом в области носогубного треугольника, двусторонним конъюнктивитом и пневмонией. У всех пациентов в ходе обследования отмечались высокие уровни АКТГ (медиана АКТГ — 246 пг/мл) и кортизола вечерней крови (медиана кортизола — 1649 нмоль/л), у всех, кроме одного, регистрировалось повышение метилированных катехоламинов в суточной моче (метанефрин — 1481 мкг/сут, норметанефрин — 735 мкг/сут). По данным методов топической диагностики (МСКТ забрюшинного пространства), у всех было выявлено образование левого надпочечника диаметром от 1,8 до 4,3 см нативной плотностью от 19 до 38 HU. Всем больным выполнена односторонняя адреналэктомия с соответствующей предоперационной подготовкой (альфа- и бета-адреноблокадой). В послеоперационном периоде развилась вторичная надпочечниковая недостаточность, вследствие чего пациенты нуждались в назначении заместительной терапии глюкокортикоидами. При морфологическом исследовании удаленных новообразований одно было представлено феохромоцитомой с выраженной кистозной трансформацией и сохранной тканью на периферии, остальные опухоли представляли собой солидные узлы и имели гистологическое строение феохромоцитомы. При иммуногистохимическом исследовании в одном наблюдении выявлена очаговая экспрессия АКТГ (около 30% клеток), в других же отмечалась интенсивная диффузная экспрессия. Индекс пролиферации Ki-67 был невысок во всех новообразованиях и не превышал 4%. Кроме того, в ткани ФХЦ выявлено наличие рецепторов соматостатина 2 или 5 подтипов (РССТ2, РССТ5), а в 1 случае — и их сочетание.

Особенностью течения заболевания у нашей пациентки является стремительное развитие гиперкортицизма (несколько месяцев) со связанными с ним тяжелыми осложнениями в виде рефрактерной гипокалиемии, сахарного диабета, обусловленного эндогенным гиперкортицизмом, снижения массы тела, миопатии, высоких уровней АКТГ и кортизола. У пациентки присутствовала классическая триада симптомов ФХЦ: высокоамплитудное повышение АД, учащенное сердцебиение и повышенная потливость. Из сопутствующих симптомов отмечались тремор и тревожное состояние, АГ стала первым признаком заболевания. Проявления ЭГ манифестировали отсроченно, только через год, что характерно для АКТГ-продуцирующей ФХЦ. Однако степень и стремительность нарастания признаков гиперкортицизма привела к тяжелому состоянию пациентки уже через 1,5 месяца после своего появления.

В данном клиническом случае наблюдалась тяжелая гипокалиемия, потребовавшая назначения не только приема таблетированных форм, но и инфузионной терапии препаратами калия. Как правило, гипокалиемия наблюдается у пациентов с первичным гиперальдостеронизмом, однако снижение уровня калия в организме может быть обусловлено тяжелым течением гиперкортицизма. По данным метаанализа Elliot PF и соавт., у пациентов с АКТГ-ЭС в ФХЦ наблюдалась гипокалиемия со средним значением калия в сыворотке крови 2,7 ммоль/л. Патогенез данного состояния обусловлен активацией минералокортикоидных рецепторов кортизолом. Уровни натрия и калия в организме регулируются альдостероном, опосредованно минералокортикоидными рецепторами почек. Сродство глюкокортикоидных и минералокортикоидных рецепторов к кортизолу равное. Тем не менее, несмотря на более высокую концентрацию кортизола в крови, чем концентрация альдостерона, минимальные колебания уровня последнего изменяют натрий-калиевый баланс. Это обусловлено действием внутриклеточного фермента 11β-гидроксистероиддегидрогеназы 2-го типа, посредством которой кортизол превращается в неактивный кортизон, предохраняя этим минералокортикоидные рецепторы от взаимодействия с кортизолом. При высоком уровне кортизола в крови данный защитный механизм нарушается. Активация минералокортикоидных рецепторов кортизолом влечет за собой увеличение АД, повышение внеклеточного объема жидкости, а также гипокалиемию [9][10]. Несвоевременная коррекция гипокалиемии может привести к различным нарушениям сердечной деятельности вплоть до внезапной смерти от остановки сердца.

Согласно литературе, у больных, страдающих от синдрома гиперкортицизма, отмечаются такие симптомы, как психическая астения, расстройства настроения в виде депрессивных или маниакальных состояний, сенестопатии, ипохондричность, снижение или полное отсутствие полового влечения, усиление аппетита и жажды, нарушение сна [17]. Хотя в литературе описана многообразная клиническая симптоматика психических нарушений при синдроме гиперкортицизма, у нашей пациентки не наблюдалось ярко выраженных симптомов, отмечались нарушения в виде тревожности, снижения памяти и способности к концентрации, депрессии, вероятнее всего, это было связано с быстрым развитием развернутой клинической картины.

Поиск источника АКТГ-продукции при наличии образований в различных эндокринных органах иногда может быть весьма затруднительным. Диагностика АКТГ-ЭС состоит из трех этапов: подтверждение гиперкортицизма, дифференцировка между АКТГ-зависимой и АКТГ-независимой формами, а также между гипофизарной и внегипофизарной секрецией АКТГ [3][6]. В данном клиническом случае, с учетом наличия у пациентки микроаденомы гипофиза, селективный забор крови из нижних каменистых синусов являлся первостепенным для определения генеза продукции АКТГ. Насколько известно, уровень гиперсекреции АКТГ пропорционален размерам аденомы гипофиза, в то время как у нашей пациентки такой взаимосвязи обнаружено не было. Данный факт позволил заподозрить другой источник избыточной секреции АКТГ. Гиперпластические процессы в надпочечниках нередки при АКТГ-эктопированном синдроме; они обусловлены выраженной стимуляцией коркового слоя надпочечников высокими уровнями АКТГ, как правило, носят диффузный характер — надпочечники увеличены за счет множественных узлов различного размера. В данном клиническом случае показано, что верификация сочетанной гормональной активности высокоплотного образования надпочечника необходима для правильной постановки диагноза и определения спектра необходимой подготовки перед операцией.

У пациентов с АКТГ-ЭС в ФХЦ особую важность имеет предоперационная подготовка. Неуправляемая гемодинамика в интра- и послеоперационном периоде у таких пациентов крайне опасна, поэтому для ее предотвращения необходимо проводить медикаментозную предоперационную подготовку, используя препарат группы селективных α1-адреноблокаторов доксазозин в дозе 4–6 мг в сутки. Подготовленность пациентов к оперативному вмешательству определяется критериями эффективности терапии: достижение целевого уровня ЧСС, нормализация АД, ликвидация индуцированного избытком катехоламинов гиповолемического синдрома. Необходимо заблаговременное назначение вышеуказанной терапии. По данным ретроспективных исследований, α-адреноблокаторы должны быть назначены минимум за 7 дней до операции [3][4].

Помимо адренергической блокады, достижение эукортицизма перед оперативным вмешательством имеет чрезвычайно важное значение, поскольку позволяет уменьшить/нивелировать метаболические, сердечно-сосудистые, костно-мышечные и др. осложнения, связанные с высоким уровнем кортизола. Согласно клиническим исследованиям, имеются данные об эффективности применения аналогов соматостатина для снижения гиперсекреции АКТГ эктопическими очагами. Так, в 2021 г. описано успешное применение октреотида пролонгированного действия у молодой женщины с тяжелым течением эктопического синдрома АКТГ [11]. Продемонстрированная данными сцинтиграфии с 99-Тс-тетроктидом гиперэкспрессия соматостатиновых рецепторов позволила предположить возможность использования препарата аналогов соматостатина для снижения секреции АКТГ. Но после введения ланреотида длительного действия уменьшения уровней АКТГ и кортизола не последовало. Такое отсутствие эффекта может быть обусловлено как высокой активностью гормональной секреции, так и отсутствием сопряженности экспрессируемых рецепторов с пострецепторными путями, управляющими секрецией АКТГ. Второй попыткой уменьшить кортизолемию было назначение блокатора стероидогенеза кетоконазола, что также не было эффективным, — при контроле кортизола суточной мочи наблюдался только рост концентрации гормона, причем экспоненциальный. Более того, на фоне принимаемой дозы ингибиторов стероидогенеза отмечалось повышение уровня печеночных трансаминаз.

На сегодняшний день основным методом лечения АКТГ-эктопии остается радикальное хирургическое лечение очага гиперпродукции [1]. В отдельных случаях требуется двусторонняя адреналэктомия. В нашем клиническом случае хирургическое лечение привело к быстрому регрессу основных симптомов и стойкой ремиссии, что позволило отменить медикаментозные препараты.

После удаления феохромоцитомы наблюдалась транзиторная надпочечниковая недостаточность с быстрым восстановлением секреции гипофизарного АКТГ и достаточной секрецией кортизола в контралатеральном надпочечнике, в связи с чем пациентка выписана без заместительной терапии глюкокортикостероидами. Вторичная надпочечниковая недостаточность, развившаяся в раннем послеоперационном периоде, является критерием радикальности проведенного оперативного вмешательства [1][2]. Вышеуказанный процесс происходит вследствие длительной чрезмерной кортизолемии, которая, блокируя гипоталамо-гипофизарнуо-надпочечниковую ось, нарушает механизм отрицательной обратной связи.

## Заключение

Представленный клинический случай демонстрирует сложность дифференциальной диагностики эндогенного гиперкортицизма с необходимостью последовательного использования диагностических проб, сложных инструментальных и инвазивных методик. Патогенетическая особенность эктопической секреции АКТГ из феохромоцитомы с созданием высоких концентраций гормона на уровне его действия в надпочечниках обусловила фульминантное развитие заболевания и его жизнеугрожающих осложнений, коррекция которых стала отдельной трудной задачей предоперационной подготовки. Относительно непродолжительное существование гиперкортицизма у пациентки не привело к подавлению обратной отрицательной связи гипоталамус-гипофиз-надпочечники, и необходимости в назначении заместительной терапии глюкокортикостероидами не было, а за 6 месяцев динамического наблюдения отмечен регресс всех осложнений заболевания.

## Дополнительная информация

Источники финансирования. Работа выполнена по инициативе авторов без привлечения финансирования.

Конфликт интересов. Авторы декларируют отсутствие явных и потенциальных конфликтов интересов, связанных с содержанием настоящей статьи.

Участие авторов. Усеинова З.Т. — сбор и обработка материала, написание статьи; Пигарова Е.А. — разработка концепции и дизайна исследования, внесение правок и одобрение финальной версии рукописи; Бельцевич Д.Г. — внесение правок и одобрение финальной версии рукописи; Шевэ А. — сбор и обработка материала, написание статьи; Дзеранова Л.К. — внесение правок и одобрение финальной версии рукописи; Ситкин И.И. — интерпретация результатов; Тарбаева Н.В. — интерпретация результатов; Дегтярев М.В. — интерпретация результатов; Хайриева А.В. — интерпретация результатов; Бондаренко Е.В. — интерпретация результатов, внесение правок в финальную версию рукописи. Все авторы одобрили финальную версию статьи перед публикацией, выразили согласие нести ответственность за все аспекты работы, подразумевающую надлежащее изучение и решение вопросов, связанных с точностью или добросовестностью любой части работы.

Согласие пациента. Пациентка добровольно подписала информированное согласие на публикацию персональной медицинской информации в обезличенной форме в журнале «Проблемы эндокринологии».
